# Supplementary material for: Evidence supported by Mendelian randomization: impact on inflammatory factors in knee osteoarthritis
Source: Front Med (Lausanne). 2024 May 28;11:1382836. doi: 10.3389/fmed.2024.1382836 (PMC11165061; doi:10.3389/fmed.2024.1382836)
Supplement: Supplementary file 1 [file Data_Sheet_1.ZIP › data/GCST90274846_5e6/846.leaveoneout.pdf]

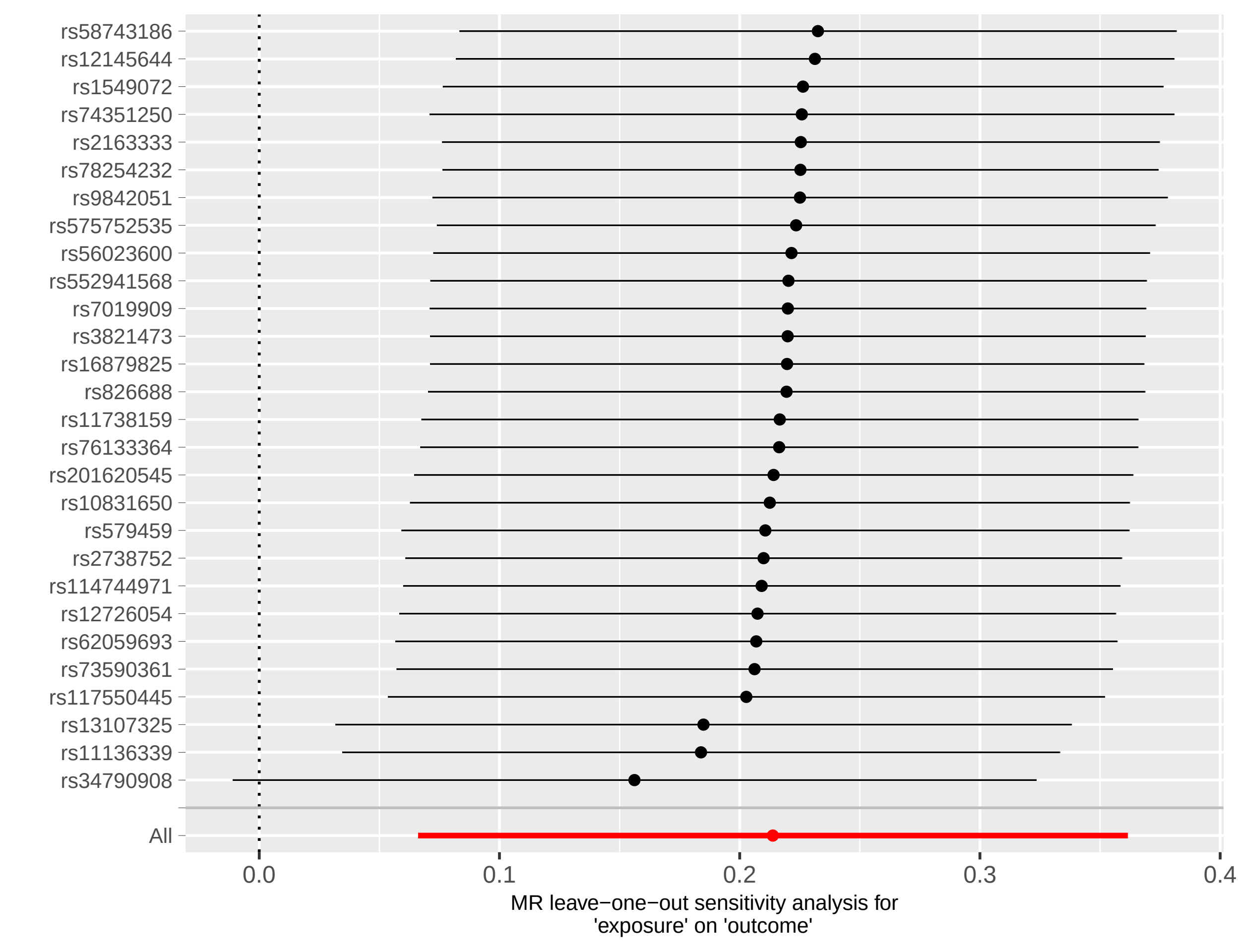
rs58743186
rs12145644
rs1549072
rs74351250
rs2163333
rs78254232
rs9842051
rs575752535
rs56023600
rs552941568
rs7019909
rs3821473
rs16879825
rs826688
rs11738159
rs76133364
rs201620545
rs10831650
rs579459
rs2738752
rs114744971
rs12726054
rs62059693
rs73590361
rs117550445
rs13107325
rs11136339
rs34790908
All
0.0
0.1
0.2
0.3
0.4
MR leave−one−out sensitivity analysis for
'exposure' on 'outcome'
